# Characterization of KPC-160, a novel Ω-loop-deleted KPC variant on a dual-copy plasmid that confers cefiderocol resistance

**DOI:** 10.1128/aac.01358-25

**Published:** 2026-05-06

**Authors:** Yuxuan Liu, Hanxu Hong, Qisen Huang, Linping Fan, Peng Liu, DanDan Wei, Yang Liu

**Affiliations:** 1 Department of Clinical Laboratory, The First Affiliated Hospital of Nanchang University 117970, Nanchang, China; 2 School of Public Health, Jiangxi Medical College, Nanchang University 568737, Nanchang, China; 3 Center for Molecular Diagnosis and Precision Medicine, The First Affiliated Hospital, Jiangxi Medical College, Nanchang University 74653, Nanchang, China; 4 Jiangxi Medical Center for Critical Public Health Events, The First Affiliated Hospital, Jiangxi Medical College, Nanchang University 74653, Nanchang, China; Entasis, Big Bay, Michigan, USA

**Keywords:** *Klebsiella pneumoniae*, KPC-160, cefiderocol, plasmid dissemination, resistance mechanism

## Abstract

The evolution of KPC-type carbapenemase variants is a major driver of resistance in *Klebsiella pneumoniae*. Cefiderocol (FDC), a next-generation siderophore cephalosporin, demonstrates potent activity against many KPC producers; however, emerging variants are compromising its efficacy. Here, we report the identification and functional characterization of a novel KPC variant, KPC-160, which confers resistance to FDC. This variant, harboring a unique two-amino acid deletion (ΔGlu167–Leu168) within the Ω-loop, was discovered in a clinical ST15-KL19 *Klebsiella pneumoniae* isolate. Antimicrobial susceptibility testing, whole-genome sequencing, and conjugation assays defined the resistance profile and genetic context, while carbapenemase activity assays, enzyme kinetics, and molecular modeling elucidated the functional mechanism. Strain K1661 exhibited FDC resistance (MIC = 8 µg/mL) alongside multidrug resistance. Genomic analysis identified the novel *bla*_KPC-160_ variant carried on an IncFIB(K) plasmid in dual IS26-flanked copies, displaying efficient transfer frequencies (3.73 × 10⁻³−4.55 × 10⁻⁵). Compared with *bla*_KPC-2_, *bla*_KPC-160_ featured a ΔGlu167–Leu168 deletion, which markedly increased FDC MICs (16-fold higher than KPC-2) and increased its affinity for FDC. Molecular docking and dynamics simulations indicated that this deletion stabilized FDC binding within the active site, thereby facilitating resistance. Conventional carbapenemase detection assays (mCIM, GeneXpert Carba-R, and NG-Test Carba 5) successfully identified KPC-160. These findings describe *bla*_KPC-160_, a deletion variant conferring clinically relevant FDC resistance via enhanced interaction and compromised inhibition. Its localization on a highly transmissible plasmid with a dual-copy architecture underscores its potential for rapid dissemination. Continuous genomic surveillance of emerging KPC variants is critical to preserving the clinical utility of FDC.

## INTRODUCTION

Carbapenem-resistant *Klebsiella pneumoniae* (CRKP) is recognized as a major global public health threat, often causing nosocomial outbreaks with high mortality (1). The resistance of CRKP largely arises from the dissemination of plasmid-borne carbapenemase genes, such as *bla_KPC_, bla_NDM_, bla_IMP_, and bla_OXA-48_* (2). Cefiderocol (FDC), a novel siderophore cephalosporin, exhibits potent activity against gram-negative pathogens, including carbapenem-resistant (CR) isolates (3, 4). By exploiting a unique “Trojan horse” strategy, FDC utilizes bacterial iron uptake systems to achieve efficient accumulation in the periplasmic space, where it exerts its bactericidal effect (5, 6). In addition, FDC demonstrates remarkable stability against a broad spectrum of β-lactamases, including both serine and metallo-carbapenemases (7).

However, it has been reported that nearly one-sixth of isolates from patients with carbapenem-resistant (CR) infections exhibit reduced susceptibility to FDC, with MICs increased by ≥4 fold (8). Reported mechanisms of FDC resistance include mutations in siderophore receptor genes (e.g., *fhuA* and *cirA*), alterations in β-lactamase activity, variations in penicillin-binding proteins (PBPs), and dysregulation of two-component regulatory systems (9–13). Although FDC is renowned for its high stability against β-lactamases, accumulating evidence indicates that β-lactamase activity may differ substantially across resistant strains. Notably, certain KPC variants such as those conferring resistance to ceftazidime/avibactam have also been associated with elevated FDC MICs. Even single- or double-amino acid substitutions in enzymes such as KPC-2, NDM-1, and CTX-M-15 can mediate resistance to FDC (13), underscoring the remarkable adaptive potential of β-lactamases.

While numerous KPC variants have been documented, the continued emergence of structurally distinct mutants with altered substrate profiles underscores the ongoing evolutionary arms race. To date, no KPC variant featuring a deletion within the critical Ω-loop region has been reported to confer elevated resistance to FDC. In this study, we describe the discovery and comprehensive analysis of KPC-160, a novel KPC variant distinguished by a two-amino acid deletion (ΔGlu167–Leu168) in the Ω-loop. Uniquely located in a dual-copy configuration on a transmissible plasmid, KPC-160 drives FDC resistance through a mechanism involving enhanced binding stability and catalytic efficiency against this drug. Our findings unveil a previously unrecognized evolutionary pathway of KPC-mediated resistance and highlight a potential challenge to the clinical efficacy of FDC. Therefore, this study addresses a critical knowledge gap by characterizing KPC-160, a novel KPC variant defined by a unique Ω-loop deletion (ΔGlu167–Leu168), which confers cefiderocol resistance. The discovery of this mechanism, coupled with its location on a transmissible, dual-copy plasmid, represents the central novelty of our work and underscores a new challenge in combating antibiotic resistance.

## MATERIALS AND METHODS

### Bacterial identification and antimicrobial susceptibility testing

The *Klebsiella pneumoniae* isolate was obtained from a clinical specimen at a tertiary care hospital in Jiangxi, China. Species identification was performed using matrix-assisted laser desorption/ionization time-of-flight mass spectrometry (MALDI-TOF MS, Bruker Daltonics). Minimum inhibitory concentrations (MICs) were determined by the broth microdilution method, with each strain tested in triplicate. Interpretations for most antibiotics followed the European Committee on Antimicrobial Susceptibility Testing (EUCAST) guidelines, whereas imipenem/relebactam and tigecycline were interpreted according to Clinical and Laboratory Standards Institute (CLSI) criteria (14). The MIC of FDC was determined using ID-CAMHB per EUCAST 2023 guidelines (https://www.eucast.org/), although it is noted that an MIC of ≥8 µg/mL is interpreted as resistant by EUCAST, but as intermediate by CLSI. *Escherichia coli* ATCC 25922 was used as the quality control strain.

### Whole-genome sequencing and bioinformatic analysis

Genomic DNA was extracted from single colonies in the logarithmic growth phase using the TIANamp Bacterial DNA Kit (TIANGEN Biotech, Beijing, China). Whole-genome sequencing was performed on both the Illumina HiSeq X Ten platform (Illumina, San Diego, CA, USA) and the PacBio Sequel II system (Pacific Biosciences, Menlo Park, CA, USA). Raw sequencing reads were quality-filtered using Trimmomatic v0.39 (15), and short-read assemblies were generated with SPAdes v3.15.4 (16). Hybrid assemblies combining short and long reads were conducted using Unicycler v0.5.0 (17). Phylogenetic trees were constructed using the maximum likelihood method and visualized and annotated with tvBOT.

### Quantitative PCR (qPCR) experiments

Total RNA was extracted using the RNAprep Pure Cell/Bacteria Kit (TIANGEN, Beijing, China). Real-time quantitative reverse transcription PCR (qRT-PCR) was performed on a LightCycler 480 system (Roche, Rotkreuz, Switzerland) using TB Green Premix Ex Taq II (Takara). The 16S rRNA gene was used as an internal reference to assess the expression levels of target antibiotic resistance genes. Relative gene expression was calculated using the 2^-ΔΔCT^ method.

For real-time quantitative PCR (qPCR), target genes were cloned into plasmid vectors, and standard curves were constructed through serial dilutions. Absolute copy numbers of each gene were determined using these standard curves. The single-copy plasmid genes *repA* and *cmR* served as quantitative references to calculate the relative amplification copy number of the *bla*_KPC_ gene (18) (primer sequences are listed in Table S1).

### Plasmid transformation, conjugation assays, and carbapenemase detection

The *bla*_KPC_ gene was cloned into the pBAD33 plasmid and transformed into *E. coli* DH5α and *K. pneumoniae* ATCC 700603. Transformants were selected on Luria-Bertani (LB) agar containing chloramphenicol (50 μg/mL), and the presence of *bla*_KPC_ was confirmed by PCR. To evaluate the transferability of cefiderocol resistance, conjugation experiments were performed using *K. pneumoniae* K1661 as the donor and *E. coli* J53 as the recipient. Transconjugants were selected on LB agar containing meropenem (1.5 μg/mL) and sodium azide (100 μg/mL) and further confirmed by PCR and S1-PFGE (primer sequences are listed in Table S1). Carbapenemase production was assessed using the modified carbapenem inactivation method (mCIM), GeneXpert Carba-R molecular detection, and NG-Test Carba 5 immunochromatographic assays.

### Protein expression and purification

The coding sequence of *bla*_KPC_ was cloned into the pET-28a vector with an N-terminal His₆ tag. The recombinant plasmid was transformed into *E. coli* BL21 (DE3) (primer sequences are listed in Table S1). Transformants were cultured overnight at 37°C in LB broth containing kanamycin (50 μg/mL). The following day, cultures were grown at 30°C to an OD₆₀₀ of 0.6–0.8 and induced with 0.5 mM isopropyl β-D-1-thiogalactopyranoside (IPTG) for 3–4 h. Cells were harvested, lysed by sonication, and centrifuged to obtain the crude enzyme extract. The supernatant was initially purified using a HiTrap Q HP anion exchange column pre-equilibrated with MES buffer and eluted with a 0–1 M NaCl gradient. Fractions containing the target protein were concentrated using Amicon Ultra-15 centrifugal filters and further purified by size-exclusion chromatography on a HiLoad 16/600 Superdex 75 pg column.

### Enzyme activity and kinetic characterization

The hydrolytic activity of purified KPC enzymes against antibiotics was measured using UV–visible spectrophotometry. Reactions were performed in phosphate-buffered saline (PBS, 1×, pH 7.4). Substrate hydrolysis was monitored at appropriate wavelengths, with substrate concentrations set across a gradient, and initial reaction rates (v₀) were recorded at each concentration. Kinetic parameters (*K_m_* and *Kcat*) were obtained by fitting the initial rates to the Michaelis–Menten equation using GraphPad Prism 10.1.2. For FDC, competitive inhibition assays were conducted to determine the inhibition constant (*Kᵢ*). Fixed concentrations of the KPC enzyme were pre-incubated with varying concentrations of FDC, followed by the addition of a fixed concentration of nitrocefin ([S] = 100 µM), and initial reaction rates were measured. Data were analyzed and fitted in GraphPad Prism 10.1.2. Relevant parameters are provided in Table S2.

### Molecular docking and molecular dynamics (MD) simulations

The KPC protein structure was predicted using Swiss-Model, and the resulting model was subjected to molecular docking analysis using AutoDock Vina (19). The chemical structure of FDC was retrieved from the PubChem database (http://pubchem.ncbi.nlm.nih.gov) and prepared using AutoDockTools, with conformational optimization for docking. Binding energies between the protein and FDC were predicted using a combination of global search and local optimization algorithms, and docking results were visualized with PyMOL.

To investigate the interactions and stability of the complex, 100 ns molecular dynamics (MD) simulations were performed using GROMACS 2020.6 (20). The protein was parameterized with the AMBER99SB-ILDN force field, while the small-molecule FDC topology was generated using Sobtop 1.0 in combination with the GAFF force field. The system was solvated in a TIP3P water box with at least 1.0 nm buffer around the protein and neutralized with appropriate ions. Energy minimization was performed, followed by 100 ps NVT and 100 ps NPT equilibration at 298.15 K. Production simulations were carried out in the NPT ensemble with a 2 fs integration step; long-range electrostatics were treated using the PME method, and trajectories were saved every 10 ps.

## RESULTS

### Clinical isolate characteristics

The *Klebsiella pneumoniae* strain K1661 was isolated from a urine specimen of a 37-year-old male inpatient with paraplegia, complicated by spinal cord injury, urinary tract infection, and urinary calculi. The patient received a 23-day course of cefoperazone/sulbactam, with amikacin added from day 8 for 16 days, and intermittent ceftazidime/avibactam. The infection was ultimately controlled, and the patient was discharged. Antimicrobial susceptibility testing showed that K1661 was resistant to cefiderocol (MIC = 8 µg/mL) and exhibited resistance to most β-lactam antibiotics (Table 1), including carbapenems (meropenem MIC = 16 µg/mL) and aztreonam (MIC ≥ 64 µg/mL). Additionally, the strain demonstrated high-level resistance to conventional cephalosporins (ceftazidime and cefotaxime) and aminoglycosides (amikacin, MIC ≥ 64 µg/mL), qualifying K1661 as a multidrug-resistant (MDR) isolate.

**TABLE 1:** Antimicrobial susceptibility profiles of clinical *Klebsiella pneumoniae* strain K1661 and KPC transformants^*a*^

Antimicrobial	MIC (μg/mL)						
	K1661	DH5α/pBAD33	DH5α/pBAD33_ KPC-2	DH5α/pBAD33_ KPC-160	ATCC 700603/pBAD33	ATCC 700603/pBAD33_ KPC-2	ATCC 700603/pBAD33_ KPC-160
FDC	8	≤0.06	0.25	4	0.125	0.5	4
IPM	4	≤0.06	2	1	≤0.06	2	1
IMR	0.5	≤0.06	0.25	0.5	0.125	0.25	0.25
MEM	16	0.125	4	1	0.25	4	2
MEV	1	≤0.06	≤0.06	≤0.06	≤0.06	0.125	≤0.06
CAZ	≥64	0.125	16	≥64	16	32	≥64
CZA	8	0.125	0.5	2	0.125	0.5	2
CXM	≥64	0.25	16	32	32	≥64	≥64
AMK	≥64	0.5	0.25	0.5	4	8	8
ATM	≥64	0.125	32	8	16	≥64	32
TGC	0.25	≤0.06	≤0.06	≤0.06	0.06	0.06	0.125

^*a*^ FDC, cefiderocol; IPM, imipenem; IMR, imipenem/relebactam; MEM, meropenem; MEV, meropenem/vaborbactam; CAZ, ceftazidime; CZA, ceftazidime/avibactam; CXM, cefuroxime; AMK, amikacin; ATM, aztreonam; TGC, tigecycline; MIC, minimum inhibitory concentration.

### Genomic characterization

Whole-genome sequencing revealed that the genome of strain K1661 consists of a 5,401,761 bp chromosome and two plasmids of 148,999 bp and 10,060 bp, respectively, and the strain belongs to the ST15-KL19 type. Antimicrobial resistance gene analysis demonstrated that, in addition to carrying *bla*_KPC-160_, K1661 harbors the β-lactamase genes *bla*_SHV-1_ and *bla*_OXA-1_, the aminoglycoside resistance gene *aac(6')-Ib-cr*, the fosfomycin resistance gene *fosA6*, as well as the efflux pump genes *oqxA*/*oqxB*. To investigate potential alternative mechanisms contributing to cefiderocol resistance in strain K1661, we specifically analyzed the following: (i) mutations in key siderophore receptor genes (e.g., *fhuA* and *cirA*), (ii) alterations in penicillin-binding proteins (PBPs) and two-component regulatory systems, and (iii) the expression of efflux pumps (*oqxA/oqxB*). Our analysis revealed no mutations in the examined siderophore receptors, PBPs, or regulatory systems. Moreover, the expression levels of *oqxA/oqxB* were comparable to those in FDC-sensitive control strains. These findings suggest that the observed resistance phenotype is not attributable to these alternative mechanisms. Importantly, we identified a novel *bla*_KPC-2_ variant, *bla*_KPC-160_, characterized by a continuous deletion of six nucleotides between positions 498 and 505, resulting in the loss of glutamic acid and leucine residues at positions 167 and 168 (Fig. S1). This gene is located on the IncFIB(K)-type plasmid p1-K1661. To date, a total of 264 KPC variants have been identified, but no studies have indicated that *bla*_KPC-160_ contributes to resistance against β-lactam antibiotics, particularly FDC. Core-genome phylogenetic analysis indicated that different KPC variants cluster into distinct evolutionary branches according to their mutation sites (Fig. S2).

**Fig 1:**
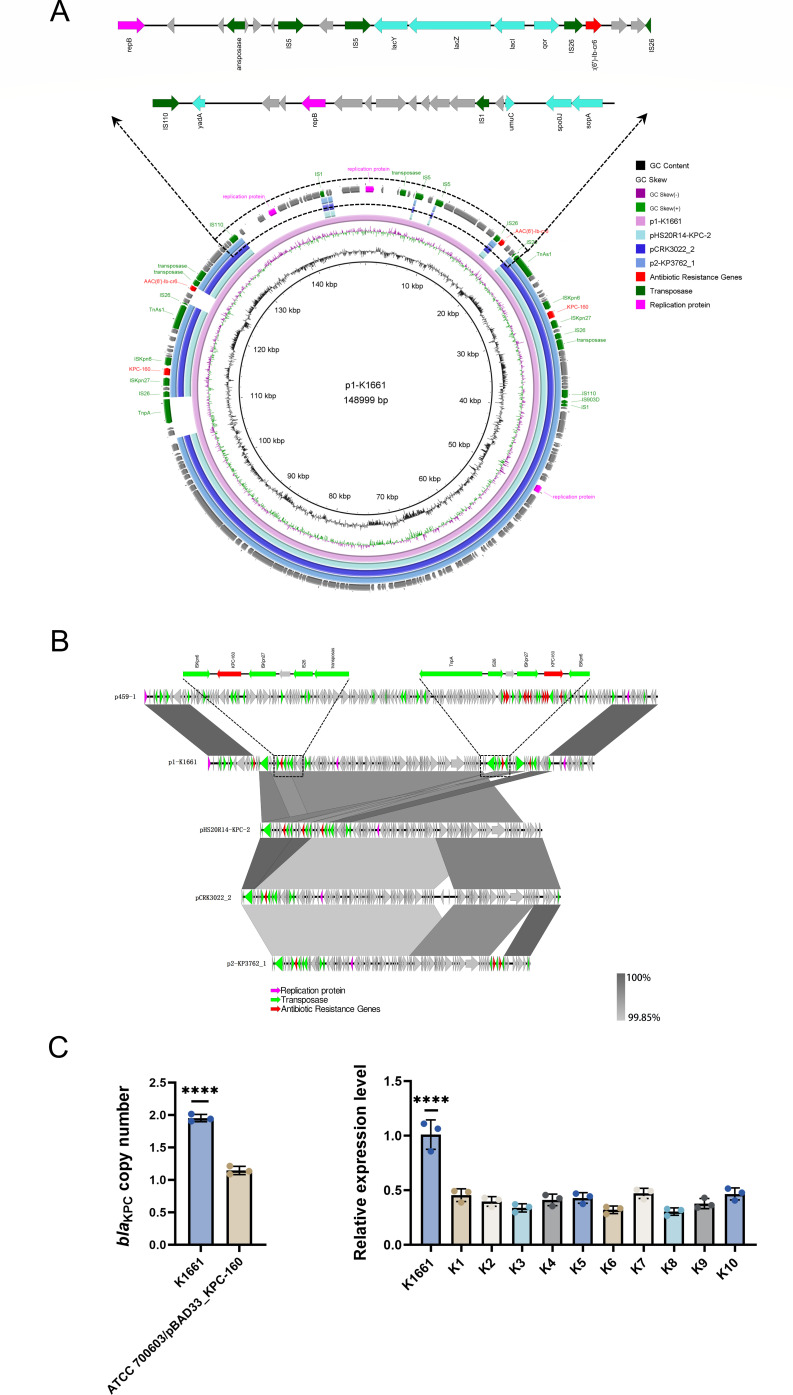
Analysis of plasmid p1-K1661 and characterization of *bla*_KPC-160_ copy number and expression. (**A**) Circular comparison of p1-K1661 with reference plasmids p2-KP3762_1, pCRK3022_2, and pHS20R14-KPC-2 from the NCBI database, with annotation of the unique 35-kb fragment in p1-K1661. (**B**) Linear comparison of p1-K1661 with p2-KP3762_1, pCRK3022_2, and pHS20R14-KPC-2, highlighting the genetic contexts of the two *bla*_KPC-160_ copies. (**C**) Analysis of the *bla*_KPC-160_ copy number in K1661 and the transformant ATCC 700603/pBAD33_KPC-160 and comparison of *bla*_KPC-160_ expression in K1661 with that in randomly selected *bla*_KPC_-positive clinical isolates (the DH5α transformant was excluded from analysis due to genetic background differences between *E. coli* DH5α and *K. pneumoniae*). ****, *P* < 0.0001.

BLAST analysis revealed that plasmid p1-K1661 shares the highest similarity with IncFII-type plasmids p2-KP3762_1 (GenBank: CP166480.1; coverage: 73%, identity: 99.96%), pCRK3022_2 (GenBank: CP091332.1; coverage: 73%, identity: 99.57%), and pHS20R14-KPC-2 (GenBank: CP064771.1; coverage: 73%, identity: 99.92%) available in the NCBI database. Comparative analysis further demonstrated that p1-K1661 exhibits high sequence homology with these reference plasmids within the multidrug resistance island carrying the *bla*_KPC-160_ gene. Notably, two copies of *bla*_KPC-160_ were identified on plasmid p1-K1661 (Fig. 1A and C), and RT-qPCR analysis showed that *bla*_KPC-160_ expression in K1661 was markedly elevated compared with other *bla*_KPC_-harboring clinical isolates (Fig. 1C).

The two *bla*_KPC-160_ copies were embedded in opposite orientations within distinct genetic contexts: one arranged as IS*26*–IS*Kpn27–bla*_KPC-160_–IS*Kpn6* and the other as TnpA–IS*26*–IS*Kpn27–bla*_KPC-160_–IS*Kpn6* (Fig. 1B). Compared with other IncFII-type *bla*_KPC-2_-bearing plasmids in the NCBI database, the TnpA element represents an additional insertion in p1-K1661, located upstream of one *bla*_KPC-160_ cassette, whereas the other cassette lacked TnpA, suggesting an IS*26*-mediated local duplication. The formation of the dual-copy configuration may involve an asymmetric amplification process mediated by IS*26* transposase activity. In contrast, approximately 35 kb of p1-K1661 exhibited no significant homology with the three reference plasmids but showed 94% coverage and 100% identity with plasmid p459-1 from *K. pneumoniae* strain Kp459 (GenBank: CP154429.1), which was also isolated from the First Affiliated Hospital of Nanchang University. This fragment harbors multiple genes implicated in plasmid recombination, stability, and dissemination (Fig. 1C). Structural genes include the plasmid replication initiation protein RepB, plasmid partitioning proteins of the ParB/RepB/Spo0J family, and SopA, suggesting independent replication and partitioning capacity. In addition, diverse mobile elements were identified, including transposases from the IS*5*, IS*26*, IS*110*, and IS*1* families; site-specific recombinases (phage integrase family); and a GIY-YIG endonuclease, underscoring the high mobility of this region. In addition, this fragment carries genes associated with lactose metabolism (*lacY*, *lacZ*, and *lacI*), the stress response gene *umuC*, and the oxidoreductase gene *qor*, which may contribute to host survival under environmental stress and support plasmid stability.

### Impact of KPC variants on antimicrobial susceptibility

We further cloned *bla*_KPC-2_ and *bla*_KPC-160_ into expression vectors and introduced them into *E. coli* DH5α and ATCC 700603. Antimicrobial susceptibility testing revealed that the FDC MIC for the transformant DH5α/pBAD33_KPC-160 was 16-fold higher than that for DH5α/pBAD33_KPC-2, with a consistent phenotype observed in the ATCC 700603 background. Compared with the recipient strain DH5α, DH5α/pBAD33_KPC-160 exhibited at least a 512-fold increase in CAZ MIC and a ≥4 fold increase relative to DH5α/pBAD33_KPC-2, whereas both transformants showed comparable MICs against CZA. Notably, KPC-160 conferred slightly reduced MICs for IPM and MEM compared with KPC-2. In contrast, novel inhibitor combinations, including IMR and MEV, retained potent activity against both KPC-2 and its variant (Table 1). We assessed the impact of curing double-copy *bla*_KPC-160_-harboring IncFIB(K) plasmid on FDC susceptibility. The results demonstrated that after plasmid curing, the susceptibility of K1661 to FDC significantly increased, with the MIC value decreasing to 0.25 μg/mL.

We further performed conjugation experiments using *E. coli* J53 as the recipient to assess the resistance level conferred by p1-K1661 and its transferability. Compared with J53, successful transconjugants exhibited at least an eightfold increase in the MIC of FDC. The conjugation frequency was calculated to be approximately 3.73 × 10⁻³–4.55 × 10⁻⁵, which was comparable to that of *bla*_KPC-2_–harboring plasmids (3.15 × 10⁻³–5.14 × 10⁻⁵), indicating that p1-K1661 has the potential to disseminate FDC resistance among *K. pneumoniae*. In addition, after serial passaging of K1661 on antibiotic plates containing sub-MIC levels of FDC for 30 generations, plasmids carrying *bla*_KPC-160_ were still stably detected, suggesting that p1-K1661 exhibits high genetic stability.

### Detection of carbapenemase activity in the KPC-160 variant

**Fig 2:**
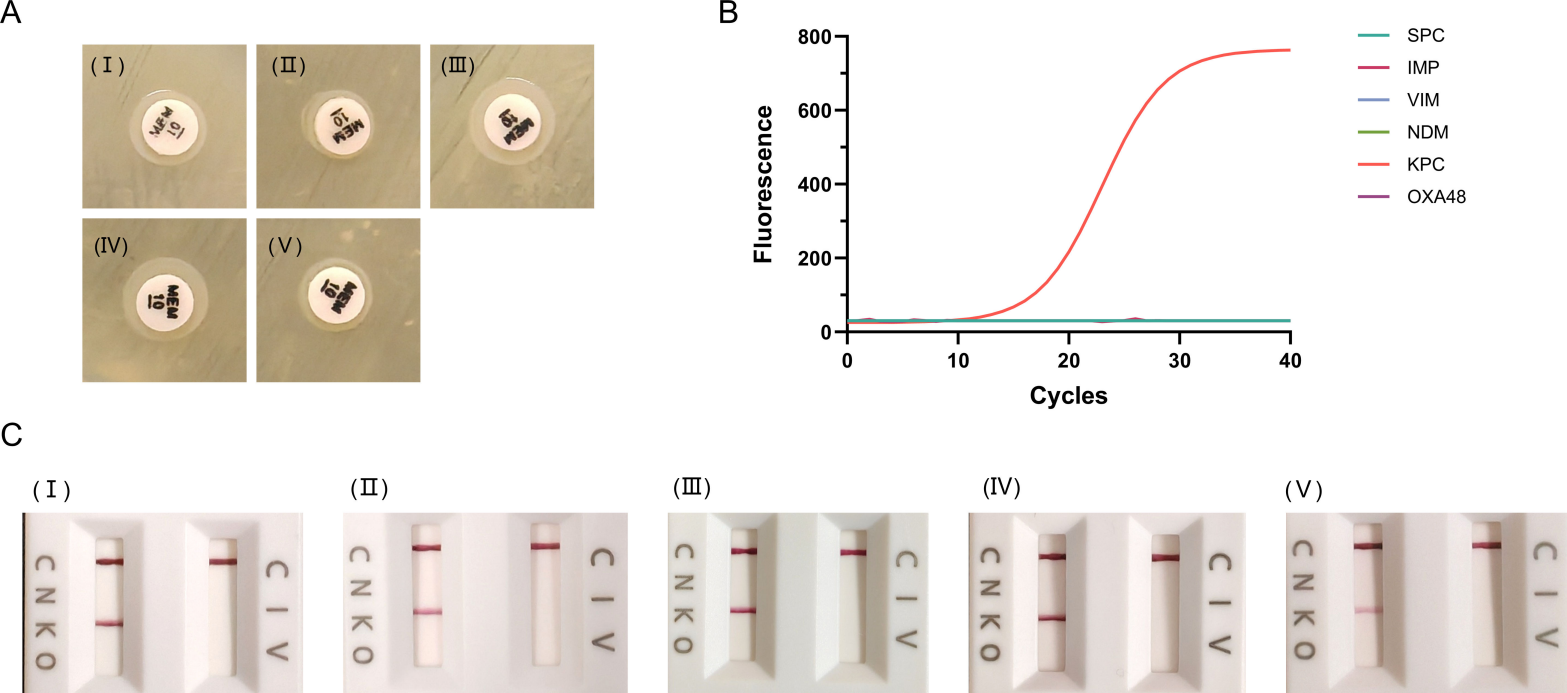
Detection of KPC-2 and KPC-160. (**A**) mCIM results showing that the inhibition zones for K1661 (I), DH5α/pBAD33_KPC-2 (II), DH5α/pBAD33_KPC-160 (III), ATCC 700603/pBAD33_KPC-2 (IV), and ATCC 700603/pBAD33_KPC-160 (VI) were all less than 6 mm. (**B**) GeneXpert Carba-R results indicating a positive *bla*_KPC-160_ signal in K1661. (**C**) NG-Test Carba 5 results showing positive detection for KPC in K1661 (I), DH5α/pBAD33_KPC-2 (II), DH5α/pBAD33_KPC-160 (III), ATCC 700603/pBAD33_KPC-2 (IV), and ATCC 700603/pBAD33_KPC-160 (VI).

To evaluate the detection capability of different assays for KPC-160 and to prevent potential clinical underdetection, carbapenemase activity was assessed using mCIM, GeneXpert Carba-R, and NG-Test Carba 5 (Fig. 2A through C). All three methods yielded positive results for KPC-160, consistent with KPC-2, in both clinical isolates and transformants. These findings indicate that KPC-160 can be accurately detected using the same approaches as for KPC-2.

### Kinetic properties and structural analysis of KPC-160 and KPC-2

Enzyme kinetic analysis revealed that, compared with KPC-2, KPC-160 exhibited significantly reduced catalytic efficiency (*Kcat*/*Km*) toward multiple β-lactam antibiotics, particularly carbapenems (e.g., MEM and IPM) and certain cephalosporins (e.g., NCF), indicating markedly diminished hydrolytic activity. In contrast, KPC-160 displayed a slightly higher *Kcat*/*Km* against CAZ than KPC-2 (0.012 vs 0.007 μM⁻¹·s⁻¹) (Table 2), although its overall hydrolytic capacity remained limited. Notably, due to the low initial hydrolytic activity of KPC-2 and KPC-160 against FDC, the hydrolytic efficiency of FDC could not be accurately determined. To evaluate the impact of KPC-160 on FDC, we used FDC as an inhibitor. The results showed that the inhibition constant (*Ki*) of FDC against KPC-2 (1.97 μM) was substantially higher than that for KPC-160 (0.36 μM), indicating a substantially lower affinity of KPC-2 for FDC than that of KPC-160.

**TABLE 2:** Kinetic parameters of KPC-2 and KPC-160^*c*^

Antimicrobial	KPC variant	*Km* (μM)	*Kcat* (s^−1^)	*Kcat/Km* (μM^−1^s^−1^)	*Ki* (μM)
NCF	KPC-2	7.06 ± 0.06	3.41 ± 0.11	0.48	/^*a*^
	KPC-160	82.97 ± 0.09	3.12 ± 1.41	0.038	/
MEM	KPC-2	162.8 ± 1.04	71.17 ± 0.94	0.44	/
	KPC-160	155.8 ± 0.18	34.58 ± 2.31	0.22	/
IPM	KPC-2	53.7 ± 0.05	22.68 ± 0.45	0.42	/
	KPC-160	115.5 ± 0.31	25.99 ± 1.41	0.23	/
CAZ	KPC-2	176.8 ± 0.62	1.24 ± 0.02	0.007	/
	KPC-160	56.87 ± 0.04	0.68 ± 0.12	0.012	/
FDC	KPC-2	ND^*b*^	ND	ND	1.97 ± 0.09
	KPC-160	ND	ND	ND	0.36 ± 0.14

^*a*^ / indicates that the value was not determined.

^*b*^ ND, not determined due to a low initial rate of hydrolysis.

^*c*^ NCF, nitrocefin; MEM, meropenem; IPM, imipenem; CAZ, ceftazidime; FDC, cefiderocol.

**Fig 3:**
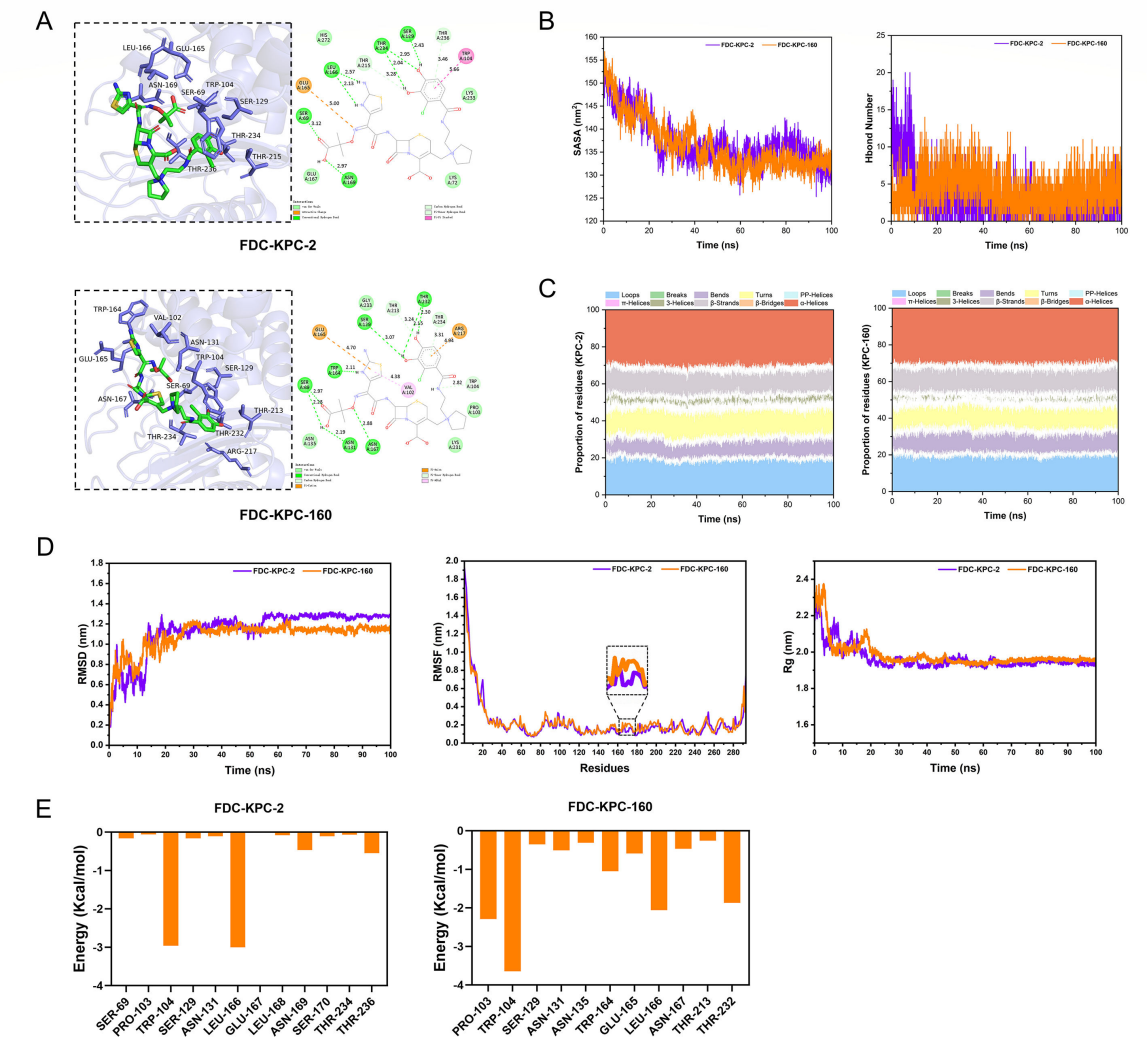
Molecular docking and molecular dynamics simulation of KPC-2 and KPC-160 with FDC. (**A**) Molecular docking showing differences in hydrogen-bond interactions of FDC within the active site pockets of KPC-2 and KPC-160. (**B**) Time-dependent changes in solvent-accessible surface area (SASA) and number of hydrogen bonds for the complexes. (**C**) Stacked area plots showing temporal changes in protein secondary structure. (**D**) Time-dependent RMSD, RMSF, and radius of gyration (Rg) of the protein–ligand complexes. (**E**) Per-residue decomposition of binding energy for key amino acid residues in the protein–ligand complexes.

To further investigate the molecular differences between KPC-2 and KPC-160, molecular docking and molecular dynamics (MD) simulations were performed. Docking results revealed differences in the binding site of FDC between KPC-2 and KPC-160. The binding energy of FDC with KPC-2 was −7.9 kcal/mol, whereas with KPC-160, it was −8.5 kcal/mol, suggesting an enhanced binding affinity of KPC-160 for FDC (Fig. 3A). During MD simulations, as shown in Fig. 3D, the FDC–KPC-2 complex stabilized at approximately 60 ns, whereas FDC–KPC-160 reached a stable state earlier at around 40 ns. The average root-mean-square deviation (RMSD) values were 1.14 nm (SD = 0.04) for KPC-2 and 1.08 nm (SD = 0.02) for KPC-160, indicating that KPC-160 exhibits greater structural stability, while KPC-2 displayed more pronounced atomic fluctuations. Root-mean-square fluctuation (RMSF) analysis revealed that the Ω-loop region (Arg164–Asp179) of KPC-160 had significantly higher fluctuations than KPC-2, potentially due to loop structural expansion induced by Ω-loop truncation, thereby enhancing local flexibility. Radius of gyration (Rg) analysis showed that KPC-160 reached structural stability earlier than KPC-2, with smaller fluctuations during the later simulation stage. Solvent-accessible surface area (SASA) analysis indicated a decreasing trend for both complexes, with FDC–KPC-160 exhibiting a higher number of hydrogen bonds in the later simulation period (1–8 vs 1–5) (Fig. 3B). Secondary structure analysis of KPC-2 and KPC-160 revealed that KPC-2 exhibited greater fluctuations in α-helices, β-strands, 3_10-helices, bends, turns, and loop structures, whereas KPC-160 showed a notable reduction in 3_10-helices and turns, accompanied by increased proportions of bends and loops (Fig. 3C), consistent with structural changes caused by the Ω-loop deletion. MM-PBSA calculations indicated that the binding free energy (ΔG_binding_) of FDC with KPC-2 and KPC-160 was −31.85 kcal/mol and −41.65 kcal/mol, respectively. Per-residue decomposition analysis revealed that the deletion of Glu167 and Leu168 in KPC-160 not only removed their direct favorable contributions to binding (Fig. 3E) but also induced adaptive rearrangements of neighboring residues within the binding pocket. For instance, residues such as Pro103, Trp164, and Thr232 exhibited significantly enhanced contributions to FDC binding in KPC-160, suggesting cooperative effects. Further dynamic cross-correlation matrix (DCCM) analysis demonstrated that the loss of Glu167 and Leu168 disrupted the original residue interaction network, promoting stable interactions between certain residues and the ligand, thereby remodeling the microenvironment of the binding cavity.

Molecular docking and molecular dynamics results indicate that, compared with KPC-2, KPC-160 exhibits stronger binding affinity and greater binding stability with FDC. The truncation of the Ω-loop increases the flexibility of the active site pocket, thereby modulating its interactions with FDC.

## DISCUSSION

Carbapenemase KPC is capable of efficiently hydrolyzing penicillins, all cephalosporins, monobactams, and carbapenems, and even exhibits partial resistance to certain β-lactamase inhibitors (21). In recent years, the prevalence of ST15 *K. pneumoniae* clones carrying KPC-2 (ST15-CRKP) has been continuously increasing in China (22). At the same time, the detection rate of KPC variants has also risen sharply, with the number of newly identified *bla*_KPC_ subtypes in the past 2 years surpassing the total discovered over the previous 2 decades (23), reflecting the rapid adaptive evolution of KPC enzymes under antibiotic selective pressure.

FDC is a novel siderophore cephalosporin. Although FDC has not yet been approved for clinical use in China, reduced susceptibility to FDC has already been reported in certain CRKP isolates from China (24–27). Our study provides experimental evidence that a deletion mutation within the Ω-loop of KPC can lead to resistance to cefiderocol, representing a novel resistance mechanism distinct from previously reported point mutations in KPC variants. The variant, KPC-160 (ΔGlu167–Leu168), not only exhibits a unique genetic signature but also resides in a dual-copy plasmid architecture, amplifying its resistance phenotype and dissemination potential. This study provides evidence that a deletion within the KPC Ω-loop (ΔGlu167–Leu168), defining the novel variant KPC-160, directly confers resistance to cefiderocol. This finding is distinct from previously reported point mutations and reveals a new evolutionary path for KPC-mediated resistance.

The β-lactamase activity of KPC enzymes relies on the catalytic serine residue located at Ambler position 70, which is surrounded by four critical structural loops: the loop between α3 and α4 helices (Leu102–Ser106), the Ω-loop (Arg164–Asp179), the loop between β3 and β4 strands (Cys238–Thr243), and the loop between the β5 strand and the α11 helix (Ala267–Ser275) (28, 29). Together, these elements constitute the active pocket of KPC enzymes, which plays an essential role in substrate recognition and hydrolysis. Therefore, mutations that alter the conformation of these regions can potentially reshape the substrate spectrum and inhibitor susceptibility. A key concern in characterizing resistance in clinical isolates is the potential confluence of multiple mechanisms. In K1661, we systematically evaluated and ruled out contributions from mutations in siderophore receptors (*fhuA and cirA*), alterations in PBPs and two-component systems, and overexpression of the *oqxA/B* efflux pump. It should be noted that in addition to the KPC-160 variant, strain K1661 harbors a truncation mutation in the *ompK35* gene. Although literature reports indicate that OmpK35 deficiency may synergistically contribute to cefiderocol resistance by reducing outer membrane permeability (30), the conclusive evidence from our functional studies—specifically, the transfer of FDC resistance via the *bla*_KPC-160_-bearing plasmid alone, coupled with the direct demonstration of enhanced binding and compromised inhibition of FDC by the purified KPC-160 enzyme—collectively establishes the ΔGlu167–Leu168 deletion in KPC-160 as the primary and sufficient determinant conferring cefiderocol resistance in this clinical strain.

In this study, we identified that the mutation site of KPC-160 is located within the Ω-loop. The deletion of Glu167 and Leu168 not only alters the local structure of the binding pocket but also remodels the microenvironment and dynamic features of the active site through an allosteric effect. Molecular docking and dynamics simulations demonstrated that this deletion promotes the formation of a tighter hydrogen-bonding network between KPC-160 and FDC, with the binding free energy (ΔG_binding_) reduced to –41.65 kcal/mol, indicating markedly enhanced binding stability. Binding free energy decomposition and dynamic cross-correlation matrix analyses further revealed that the deletion did not merely induce local structural contraction but reconfigured the overall topology of the interaction network, thereby enhancing the stable contributions of specific residues (e.g., Pro103, Trp164, and Thr232) to ligand binding. Concurrently, the truncation of the Ω-loop increased conformational fluctuations in this region, potentially providing greater flexibility of the active pocket during substrate entry and positioning, which in turn improved the binding efficiency with FDC. This structural alteration highlights that even subtle modifications of the Ω-loop can profoundly affect the inhibitor-binding properties and resistance phenotype of KPC enzymes. Unlike certain KPC variants (e.g., KPC-33 and KPC-39), which acquire resistance to CZA through point mutations, KPC-160 exhibited little change in susceptibility to CZA or IMR but instead demonstrated enhanced hydrolytic activity against FDC. This shift in the substrate profile, accompanied by reduced activity against carbapenems, represents a “functional trade-off” strategy (31).

In addition, we identified that the dual-copy KPC-160 was located on the transferable IncFIB(K) plasmid p1-K1661, which exhibited strong horizontal transfer ability, with a conjugation frequency ranging from 10⁻³ to 10⁻⁵, indicating a high dissemination potential. Notably, *bla*_KPC_ genes are most commonly found on IncFII, IncX, and IncN plasmids (32–34), whereas IncFIB(K) replicons are more frequently associated with virulence plasmids, often carried by hypervirulent *K. pneumoniae* harboring virulence determinants such as *iuc* and *rmpA* (35), or coexisting with *bla*_NDM_ and extended-spectrum β-lactamase (ESBL) genes (36–39). The occurrence of *bla*_KPC-160_ in an IncFIB(K) replicon background is relatively rare, suggesting potential recombination or fusion events between replicons. Within this plasmid, the two copies of *bla*_KPC-160_ were embedded in distinct genetic environments, namely, IS*26*–IS*Kpn27–bla*_KPC-160_–IS*Kpn6* and TnpA–IS*26*–IS*Kpn27–bla*_KPC-160_–IS*Kpn6*, both representing typical KPC transposon modules. The presence of TnpA and multiple IS elements suggests high plasticity and mobility of these structures, which may provide the genetic basis for the ongoing evolution and dissemination of *bla*_KPC-160_. The detection of insertion sequences such as IS*26*, IS*Kpn27*, and IS*Kpn6* implies transposon-mediated duplication or amplification events (40, 41). Importantly, the dual-copy configuration may enhance the expression of *bla*_KPC-160_, thereby conferring a higher level of resistance to FDC in the host strain.

Of particular concern, although p1-K1661 shares high homology with typical KPC-2-positive plasmids and retains the conserved resistance backbone of KPC plasmids, we identified an approximately 35-kb unique fragment within p1-K1661 that was highly identical to plasmid p459-1 from an FDC-resistant *K. pneumoniae* isolate obtained from the First Affiliated Hospital of Nanchang University. This finding suggests that the fragment may have been acquired via homologous recombination or IS element-mediated integration, thereby enabling interplasmid transfer and subsequent horizontal dissemination among clinical strains in the region, playing a critical role in the local epidemiology of resistance plasmids. Similar phenomena have been reported previously. For example, Zhang et al. (42) demonstrated that mobilizable plasmids in *K. pneumoniae*, even when lacking a complete conjugation transfer module, could be co-transferred with the aid of helper plasmids, thereby facilitating the spread of multiple resistance and virulence determinants across different strains. These observations imply that the 35-kb unique fragment, with its features of autonomous replication, partitioning, and enrichment in mobile genetic elements, may allow cross-strain transfer in the presence of auxiliary plasmids. This could accelerate the regional dissemination of resistance determinants. Moreover, the structural acquisitions within this fragment may not only enhance plasmid stability but also confer broad host adaptability and higher replication efficiency, facilitating its dissemination within clinical bacterial populations. Importantly, accumulating evidence indicates that certain IncFIB(K) fusion plasmids possess the capacity to simultaneously harbor both virulence and resistance determinants, thereby constituting so-called “hybrid resistance–virulence platforms” (43–45). The variable resistance gene content, mobile element composition, and transfer-associated functions observed in p1-K1661 suggest that enhanced modular plasticity may increase its ability to disseminate resistance phenotypes across diverse hosts. This, in turn, raises the risk of cryptic dissemination of multidrug-resistant strains in general wards and even primary care settings, as well as the potential emergence of CR-hvKP hybrid plasmids, underscoring the need for ongoing surveillance and monitoring.

Compared with wild-type KPC enzymes, most reported KPC variants exhibit markedly reduced hydrolytic activity against carbapenems (46), a finding that is also supported by the enzymatic data in this study. However, KPC-160 demonstrated substantially enhanced hydrolytic activity toward FDC, resulting in a 16-fold increase in the MIC compared with KPC-2, reflecting a pronounced substrate spectrum shift driven by the deletion mutation. Moreover, despite being a structural variant, KPC-160 could still be reliably detected by mainstream assays such as mCIM, GeneXpert Carba-R, and NG-Test Carba 5, in sharp contrast to certain point-mutation variants such as KPC-31, KPC-33, and KPC-39, which have been associated with false-negative results (47, 48). These findings suggest that current diagnostic methods remain generally robust for KPC-160 detection; however, as the diversity and structural heterogeneity of KPC variants continue to expand, challenges regarding assay sensitivity and standardization are likely to intensify. There is thus an urgent need to develop variant-specific detection strategies and to integrate both phenotypic and genotypic approaches to enhance diagnostic accuracy.

This study has certain limitations as the dual-copy KPC-160 was identified in only a single isolate, and whether it exhibits a regional dissemination trend requires larger-scale epidemiological investigations. In summary, KPC-160 represents a novel KPC variant carrying a critical deletion mutation, characterized by enhanced hydrolytic activity against FDC and transmissible via efficient plasmid transfer. This discovery not only reveals a new evolutionary trajectory by which KPC enzymes adapt to FDC but also poses emerging challenges for the clinical application of FDC. To mitigate the risk of potential dissemination, early implementation of enhanced genetic screening for KPC variants, plasmid structural surveillance, and inter-institutional monitoring is warranted.

## Data Availability

Complete sequences of the *K. pneumoniae* isolate have been deposited in NCBI under BioSample no. SAMN52642891.

## SUPPLEMENTAL MATERIAL

Supplemental material aac.01358-25-s0001.docx Tables S1 and S2; Fig. S1 and S2.

ASM does not own the copyrights to Supplemental Material that may be linked to, or accessed through, an article. The authors have granted ASM a non-exclusive, world-wide license to publish the Supplemental Material files. Please contact the corresponding author directly for reuse.
