# Supplementary material for: Characterization of KPC-160, a novel Ω-loop-deleted KPC variant on a dual-copy plasmid that confers cefiderocol resistance
Source: Antimicrob Agents Chemother. 2026 May 6;70(6):e01358-25. doi: 10.1128/aac.01358-25 (PMC13231912; doi:10.1128/aac.01358-25)
Supplement: Supplemental material — Tables S1 and S2; Fig. S1 and S2. [file aac.01358-25-s0001.docx]

**Supplementary table 1.** Primers uses in the study

| **Name of primers** | **Sequence （5’-3’）** | **Name of primers** | **Sequence （5’-3’）** |
| --- | --- | --- | --- |
| 16sRNA-F | ATCGAGGAACGGAACCAACC | *cmR-F* | AACTGCCGGAAATCGTCGTG |
| 16sRNA-R | GTGGATTCATCGCAGTTGCC | *cmR-F* | CGTTCAGCTGGATATTACGGCCT |
| *bla*_KPC_-F | GGCAGTCGGAGACAAAACC | pBAD33-KPC-F | ATGCCTGCAGGTCGAATGTCACTGTATCGCCGTCTAGT |
| *bla*_KPC_-R | CCCTCGAGCGCGAGTCTA | pBAD33-KPC-R | CCGGGGATCCTCTAGTTACTGCCCGTTGACGCCCA |
| *repA-F* | CAGCGCTCGATTACCAATATGGC | pET28a-KPC-F | GGATCCGAATTCCATCATCATCATCACCATCACCACCACCACATGTCACTGTATCGCCGTCTAGT |
| *repA-R* | ACCACAGCCACCTCAGAAACG | pET28a-KPC-R | CCGCAAGCTTGTCGACTTACTGCCCGTTGACGCCC |

**Supplementary table 2.** Extinction coefficients and wavelengths used for various substrates

| Substrates | Δε (M^-1^ cm^-1^) | λ (nm) |
| --- | --- | --- |
| Nitrocefin | 17400 | 482 |
| Ceftazidime | -11300 | 260 |
| Imipenem | -9000 | 300 |
| Meropenem | -6500 | 300 |


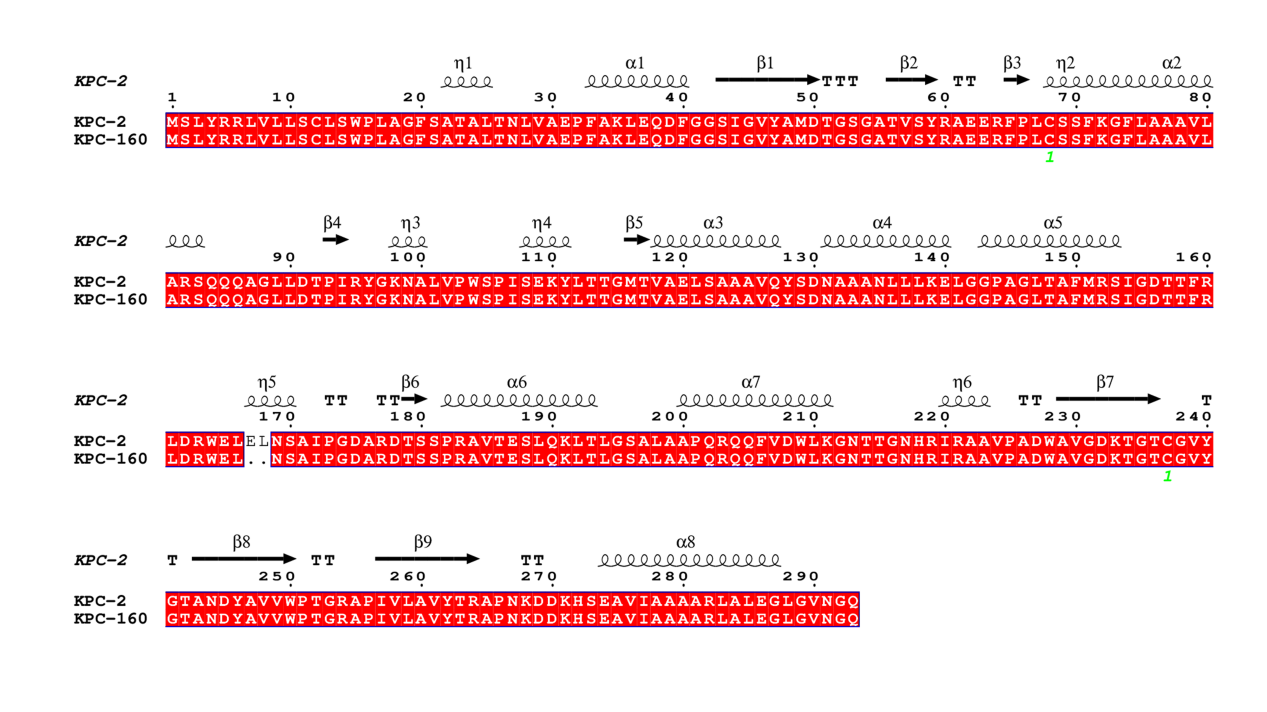


**Supplementary Figure 1. Amino acid sequence alignment of KPC-2 and KPC-160, showing that KPC-160 harbors a consecutive deletion of two amino acids (Glu167 and Leu168) compared with KPC-2.**


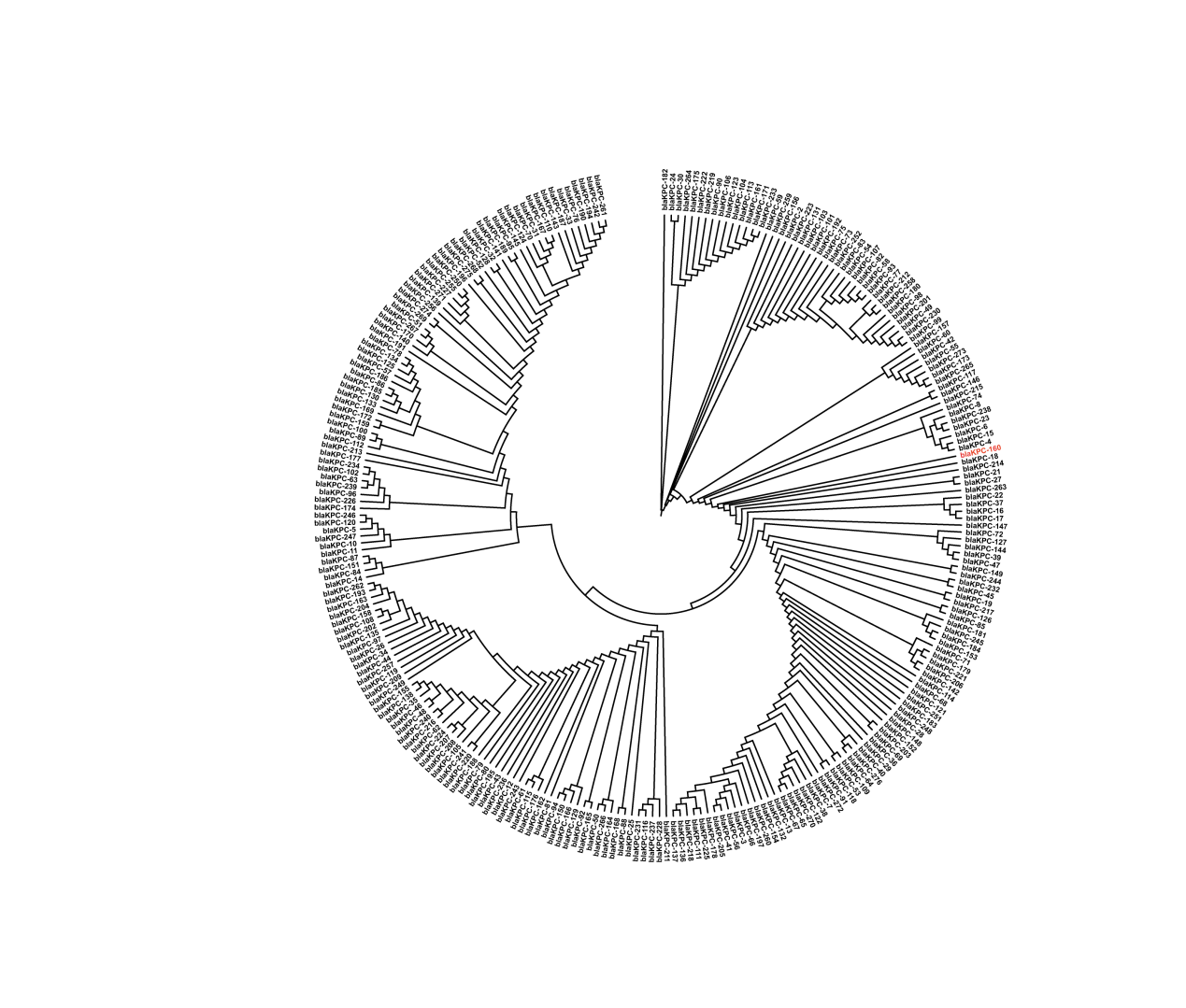


**Supplementary Figure 2.** Phylogenetic tree of KPC-160 and KPC variants. Red background denotes FDC resistance.
